# Correlates of Elder Abuse in São Paulo, Brazil: The Roles of Age, Depression, and Religion

**DOI:** 10.1007/s10943-026-02722-2

**Published:** 2026-07-09

**Authors:** José Tadeu Nunes Tamanini, Carlos Eduardo Rossi Barreto, Lucas Henrique da Silva Souza, Paulo Vitor da Costa e Silva, Sérgio José da costa, Jair Lício Ferreira Santos, Yeda Aparecida de Oliveira Duarte, Marair Gracio Ferreira Sartori, Maria Augusta Tezelli Bortolini, Rodrigo Aquino Castro

**Affiliations:** 1 Department of Medicine, Federal University of São Carlos, São Carlos, Brazil; 2 Federal Institute of Education, Science and Technology of Tocantins, Palmas, Brazil; 3 Faculty of Medicine of Ribeirão Preto, Department of Social Medicine, University of São Paulo, São Paulo, Brazil; 4 Department of Medical-Surgical Nursing, Nursing School, University of São Paulo, São Paulo, Brazil; 5 Section of Urogynecology and Pelvic Surgery, Department of Gynecology, Federal University of São Paulo, São Paulo, Brazil

**Keywords:** Elder abuse, Associated factors, Depression, Religion, SABE Study, Epidemiology

## Abstract

Elder abuse (EA) is a major public health concern worldwide, yet population-based data from low- and middle-incomecountries remain limited. This study was conducted to investigate the association factors of abuse among olderindividuals. We analyzed data from a population-based study of 1,224 community-dwelling adults aged 60 years orolder from the 2015 wave of the SABE (Health, Well-Being and Aging) Study in São Paulo, Brazil. Participants werecategorized into two groups: abused and not abused, based on responses to a validated questionnaire addressingpsychological, physical, and fi nancial abuse. With weighted analysis, the Rao-Scott test was applied to understandthe characteristics of the elderly population. Subsequently, multivariate logistic regression analysis was conducted toidentify associations between elder abuse and sociodemographic and clinical characteristics. The EA prevalence was11.8% overall (8.9% in men and 14.1% in women). In adjusted models, EA was inversely associated with age—olderparticipants were less likely to report abuse—and positively associated with depressive symptoms and Evangelicalreligious affi liation. No signifi cant diff erence was found between sexes. These fi ndings suggest that bothpsychological vulnerability and social context infl uence the risk of EA among urban older adults. Integrating mentalhealth screening and culturally sensitive strategies, including collaboration with religious communities, maystrengthen prevention and early detection eff orts in aging populations.

## Introduction

The global population is aging at an unprecedented rate, radically shifting demographic structures and expanding the cohort of adults aged 60 years and older. Age-related declines in physical and cognitive capacities, combined with social prejudice, ageism, and structural inequalities, may heighten vulnerability to elder abuse (EA). This phenomenon manifests primarily as psychological, physical, and financial maltreatment, often occurring within relationships where there is an expectation of trust. Consequently, global interest in understanding the risk factors for EA has intensified, particularly regarding family settings where spouses or adult children are frequently identified as the primary perpetrators (Pabón-Poches, 2026).

The World Health Organization (WHO) defines EA as a single or repeated act, or lack of appropriate action, occurring within any relationship where there is an expectation of trust, which causes harm or distress to an older person—a vulnerability that was noticeably exacerbated during the COVID-19 pandemic. EA has been robustly linked to severe outcomes, including major depression, increased hospitalization rates, diminished quality of life, and excess mortality, thereby imposing wide-ranging consequences on both individuals and healthcare systems (World Health Organization, 2025).

Scientific inquiry into violence against older adults dates back to 1975, when the first paper addressing EA was published in the British medical literature under the term “granny-battering.” In this seminal publication, the author noted: “I have personal knowledge of cases in which patients have been battered by relatives before admission to hospital and in which there has been no doubt that the battering was deliberate” (Burston, 1975).

Despite its long history as a clinical concern, robust prevalence estimates remain limited in low- and middle-income countries (LMICs). Nevertheless, a recent global meta-analysis indicates that approximately one in six older adults worldwide experience some form of EA (Kitaw et al., 2025). National and regional rates vary widely depending on operational definitions and methodologies, ranging from 7 to 10% in the United States, exhibiting broad variability across Europe, and hovering around 10% in the state of São Paulo, Brazil (de Andrade et al., 2025; dos Santos et al., 2022; Drommi et al., 2025; Yon et al., 2017). Despite the high prevalence and profound health impacts of EA, few population-based studies have rigorously investigated its correlates in Latin America using representative urban samples.

São Paulo—the largest metropolis in South America—presents marked socioeconomic heterogeneity and a long-standing framework for population surveillance through the SABE Study, providing a unique opportunity to examine EA in a diverse urban environment. Because the SABE Study samples community-dwelling older adults exclusively, institutionalized or hospitalized populations are not represented. Crucially, previous epidemiological research has seldom examined how religious affiliation interacts with social or psychological vulnerabilities to influence EA risk, and evidence remains scarce regarding distinct patterns across specific age subgroups (de Bernardin Gonçalves et al., 2023). Utilizing cross-sectional population-based data allows for the identification of demographic and psychosocial correlates of EA, establishing a baseline to generate hypotheses for future longitudinal research.

Therefore, this study aimed to determine the factors associated with EA among community-dwelling older adults in São Paulo, Brazil. Particular analytical attention was devoted to the "younger-old" cohort (60–69 years), who may experience distinct exposure patterns within evolving family environments. For the purposes of this investigation, EA refers specifically to verbal, psychological, physical, and financial abuse (excluding neglect and self-neglect), in alignment with the validated screening instrument applied. Furthermore, this research aligns with the United Nations Sustainable Development Goals (SDGs), directly contributing to Goal 3 (Good Health and Well-being) by addressing the mental health and safety of older populations, and Goal 16 (Peace, Justice and Strong Institutions) by identifying correlates of violence to foster protective, secure environments for vulnerable groups.

## Methods

### Study Design

This cross-sectional, household-based epidemiological study was conducted using data from the SABE (Saúde, Bem-Estar e Envelhecimento/Health, Well-Being and Aging) database, a multicenter study coordinated by the School of Public Health at the University of São Paulo (Vieira et al., 2023). Participants were initially interviewed in 2000 and subsequently followed in 2006, 2010, and 2015. Sampling weights were applied in each wave to ensure demographic representativeness by age, sex, and calendar year (Lebrão et al., 2019).

### Subjects

The SABE Study cohort comprised community-dwelling individuals aged 60 years or older, of both sexes, residing in urban areas of São Paulo, who were selected through a multistage cluster sampling design (Vieira et al., 2023).

### Data Collection and Exclusion Criteria

Data were collected through face-to-face interviews conducted by trained personnel using standardized street maps and structured questionnaires (Lebrão et al., 2019). Participants with missing data for any variables of interest were excluded from the final multivariate analysis.

### Outcome Measures

#### Dependent Variable

Elder abuse (EA) was the primary outcome. Operationally, EA is defined as a single or repeated act, or lack of appropriate action, occurring within any relationship where there is an expectation of trust, which causes harm or distress to an older person (World Health Organization, 2025). While this study adopts the World Health Organization’s comprehensive conceptual definition—which encompasses both acts of commission and omission—the specific screening instrument utilized in the SABE Study focuses exclusively on acts of commission (verbal, psychological, physical, and financial abuse) rather than neglect.

EA was assessed using items from the Geriatric Mistreatment Scale, a validated questionnaire demonstrating robust reliability (Cronbach’s alpha = 0.83) and high content validity (80%) for the geriatric population (Giraldo-Rodríguez & Rosas-Carrasco, 2013). Sexual abuse, systemic neglect, and self-neglect were not assessed by this instrument.

The screening tool consists of seven specific questions addressing experiences within the preceding 12 months: (1) “In the last year, have any of your friends or family shouted at you for no reason?”; (2) “In the last year, have any of your friends or family called you a name that you don’t like?”; (3) “In the last year, have any of your friends or family touched or spent your money without your permission?”; (4) “In the last year, have any of your friends or family threatened you because you did not do what they wanted?”; (5) “In the last year, have any of your friends or family hit or slapped you?”; (6) “In the last year, have any of your friends or family shaken you?”; and (7) “In the last year, have any of your friends or family stolen your money or something else belonging to you?”.

Response options were categorized as “yes,” “no,” or “don't know.” An affirmative response to any of the seven questions indicated the presence of EA. For sub-analysis, questions 1 and 2 were classified as verbal abuse; questions 3 and 7 as financial abuse; question 4 as psychological abuse; and questions 5 and 6 as physical abuse (Giraldo-Rodríguez & Rosas-Carrasco, 2013).

### Independent Variables

Sociodemographic characteristics included sex (male, female); age groups (60–64, 65–69, 70–74, 75–79, and ≥ 80 years); formal education level (elementary, high school, or college); income (categorized based on tertiles of the national minimum wage); and household density (1, 2, 3, or ≥ 4 people living at home).

Religious affiliation was categorized as Catholic, Protestant (Historical Protestant), Evangelical (Pentecostal and Neo-Pentecostal), Spiritualist, Other, or Atheist/Agnostic. Within this framework, the Evangelical category comprises a diverse spectrum of religious movements. In accordance with established sociological classifications of the Brazilian religious landscape, a distinction is recognized between Pentecostal denominations (referring to historical, first-wave movements with a traditional emphasis on charismatic gifts) and Neo-Pentecostal churches (referring to third-wave movements often characterized by prosperity theology and a prominent media presence).

Clinical variables included functional and cognitive markers. Functional impairment compatible with dementia was evaluated using the Pfeffer Functional Activities Questionnaire, where a score above 5 indicated significant impairment (Monteiro & Borges, 2023). Cognitive decline was assessed using the Mini-Mental State Examination (MMSE) adapted for the Brazilian population, with scores ranging from 0 to 19, and cognitive decline indicated by scores ≤ 12 (Maduro et al., 2025). Depressive symptoms were measured using the 15-item Geriatric Depression Scale (GDS), validated for Portuguese, where a score ≥ 6 indicated symptoms suggestive of depression (Sguerri et al., 2025). Physical limitation was defined by self-reported difficulty in performing basic activities of daily living (BADL) (yes/no) (Aliberti et al., 2022). History of falls was defined as the self-reported occurrence of any fall within the previous 12 months (yes/no). Comorbidities were treated as a categorical variable based on the total count of chronic conditions (0, 1–2, or ≥ 3) out of a list including hypertension, heart disease, stroke, diabetes mellitus, chronic lung disease, osteoarticular disease, cancer, and psychiatric or neurological disorders.

### Statistical Analysis

Descriptive statistics were calculated using means and standard deviations for continuous variables, and proportions for categorical variables. Population sampling weights were applied to all statistical analyses to account for the complex survey design. Bivariate associations were evaluated using the Rao–Scott chi-square test. Subsequently, multivariate logistic regression models were executed to identify independent factors associated with EA. All statistical analyses were performed using STATA software, version 14 (StataCorp, College Station, TX, USA). Statistical significance was pre-established at *p* < 0.05.

### Ethical Aspects

All participants or their legal representatives provided written informed consent prior to data collection. The study protocol ensured strict data anonymity and was approved by the Human Research Ethics Committee of the Faculty of Public Health at the University of São Paulo, Brazil (protocol number: 47683115.4.0000.5421/2015).

## Results

The present analysis incorporated data from the 2015 wave of the SABE Study, yielding a final analytical sample of 1,224 community-dwelling older adults (Table 1).

**Table 1:** Participant demographic characteristics (*N* = 1,224)

Characteristics		*N* (%)
Gender	Female	796 (65.1)
	Male	428 (34.9)
Age Group	60–69	676 (58.2)
	≥ 70 years	548 (41.8)
Educational Attainment	Elementary level	743 (68.2)
	Beyond elementary level	481 (31.8)
Personal Income	Below the national minimum income wage	340 (31.7)
	Equal to or above the national minimum wage	884 (68.3)
Interview type	Self-reported	1,110 (90.7)
	Proxy respondent	114 (9.3)

The overall prevalence of elder abuse (EA) in the population was 11.8%, presenting a significant variation between sexes: 8.9% among men and 14.1% among women (*p* = 0.005). Characterized by abuse type, the most frequently reported adverse experience was being shouted at for no reason (10.3%), which was operationally classified as verbal abuse.

In the bivariate analysis (Table 1), the prevalence of EA correlated significantly with several baseline characteristics. Specifically, higher rates of reported abuse were observed among female participants (*p* = 0.005), individuals within the younger-old cohort aged 60–69 years (*p* = 0.016), individuals screening positive for depressive symptoms (*p* < 0.001), and those reporting an Evangelical religious affiliation (*p* = 0.002). Conversely, no statistically significant bivariate associations were detected between EA and education, income, household density, functional impairment compatible with dementia, cognitive decline, physical limitations, history of falls, or the cumulative number of chronic comorbidities.

In the final multivariate logistic regression model (Table 2), which adjusted for all potential covariates and accounted for the complex survey sampling weights, age, religious affiliation, and depression emerged as independent correlates of EA.

**Table 2:** Bivariate analysis of elder abuse (EA) prevalence according to sociodemographic and clinical characteristics.

Variables	Categories	No EA (%)	EA (%)	*p*-value^*#^
Sex	Male	91.1	8.9	0.005
	Female	85.9	14.1	
Age Group	60–64	84.0	16.0	0.016
	65–69	87.2	12.8	
	70–74	88.3	11.7	
	75–79	94.0	6.0	
	80 +	93.1	6.9	
Income (Terciles)	1st	87.1	12.9	0.130
	2nd	87.9	12.1	
	3rd	91.4	8.6	
Schooling	Primary	88.5	11.5	0.577
	High School	88.8	11.2	
	College	91.4	8.6	
Religion	Catholic	90.3	9.7	0.002
	Protestant			
(Historical)	79.4	20.6		
	Evangelical (Pentecostal/Neo-Pentecostal)	81.4	18.6	
	Spiritualist	89.9	10.1	
	Other	97.3	2.7	
	Atheist	81.5	18.5	
People living at Home	1	87.6	12.4	0.123
	2	86.3	13.7	
	3	92.3	7.7	
	4 +	87.1	12.9	
Dementia	Yes	90.0	10.0	0.597
	No	88.1	11.9	
Cognitive Decline	Yes	88.0	12.0	0.921
	No	88.2	11.8	
Depression	Yes	80.8	19.2	< 0.001
	No	89.6	10.4	
Physical Limitation	Yes	89.2	10.8	0.536
	No	87.7	12.3	
Falls	Yes	85.3	14.7	0.080
	No	89.3	10.7	
Presence of comorbidities	0	90.5	9.5	0.093
	1–2	89.7	10.3	
	3 +	85.4	14.6	

Municipality of São Paulo, SP, Brazil. SABE Study, 2015 (*n* = 1,213)

^*^p < 0.05. ^#^Rao-Scott test

*n* = 1,213 represents the analytical sample with complete data for the variables presented in this table (total sample *N* = 1,224)

Individuals aged 75–79 years and those aged 80 years or older exhibited significantly lower odds of experiencing EA compared to the youngest reference cohort aged 60–64 years (OR = 0.31; 95% CI [0.14, 0.66]; *p* = 0.003 and OR = 0.39; 95% CI [0.20, 0.75]; p = 0.005, respectively). In contrast, Evangelical affiliation and mental health indicators were positively associated with the outcome. Self-reported Evangelicals were nearly twice as likely to experience EA compared to their Catholic counterparts (OR = 1.97; 95% CI [1.32, 2.93]; *p* = 0.001). Furthermore, the presence of clinically significant depressive symptoms increased the likelihood of experiencing elder abuse by approximately 71% (OR = 1.71; 95% CI [1.12, 2.59]; *p* = 0.012). No other sociodemographic or clinical covariates retained statistical significance in the fully adjusted model (Table 3).

**Table 3:** Multivariate logistic regression analysis of factors associated with Elder Abuse in a sample of older adults.

Variables	Categories	Odds Ratio	*p*-value	95% CI (Lower)	95% CI (Upper)
Sex	Female	1 (Ref)	–	–	–
	Male	0.68	0.066	0.46	1.02
Age Group	60–64	1	–	–	–
	65–69	0.75	0.296	0.44	1.28
	70–74	0.62	0.169	0.31	1.22
	75–79	0.31	0.003	0.14	0.66
	80 +	0.39	0.005	0.20	0.75
Religion	Catholic	1	–	–	–
	Protestant	2.60	0.186	0.62	10.78
	Evangelical	1.97	0.001	1.32	2.93
	Spiritual	0.79	0.572	0.35	1.77
	Other	0.22	0.129	0.03	1.56
	Atheist	1.90	0.108	0.86	4.19
Depression	No	1	–	–	–
	Yes	1.71	0.012	1.12	2.59

Municipality of São Paulo, SP, Brazil. SABE Study, 2015 (*N* = 1,185)

^*^CI: Confidence Interval. *p* < 0.05

## Discussion

This cross-sectional population-based study identified depressive symptoms and an Evangelical religious affiliation as independent correlates of elder abuse (EA), whereas increasing age demonstrated a significant inverse association with the risk of maltreatment. The overall prevalence of EA (11.8%) among community-dwelling older adults in São Paulo is highly comparable to global epidemiological estimates, although distinct local cultural, territorial, and socioeconomic frameworks likely shape its presentation. These findings underscore the complex, intersectional nature of vulnerability in late life and emphasize the urgent need for multidisciplinary preventive frameworks.

The observed prevalence aligns closely with findings from international meta-analyses indicating that approximately one in six older adults worldwide experience some form of mistreatment (Yon et al., 2017). However, our estimate is slightly lower than certain global aggregates (15.7%) or recent national data from high-income contexts such as Australia (14.8%) (Kitaw et al., 2025; Qu et al., 2024). Variations in operational definitions, cultural normalization of specific behaviors, and varying levels of social awareness regarding elder rights may explain this heterogeneity. Furthermore, underreporting remains a systemic challenge in EA research, particularly when the perpetrators are cohabiting family members or primary caregivers upon whom the older victim depends, which frequently induces feelings of shame, fear of institutionalization, or retaliation (Storey, 2020).

In the fully adjusted multivariate model, sex did not emerge as a statistically significant correlate of EA, despite a higher raw prevalence observed among women in the descriptive analysis. This discrepancy is frequent in the literature; while bivariate models often reflect gendered patterns of economic dependency, social isolation, and differential reporting behaviors, these effects are frequently attenuated when adjusting for clinical variables such as depression (Hancock & Pillemer, 2022).

Conversely, the significant inverse association between age and EA reveals that the "younger-old" cohort (60–69 years) faces a higher risk of maltreatment than the oldest-old. This pattern is consistent with prior studies indicating that younger-old individuals are more likely to remain economically active, manage family conflicts directly, and cohabit with adult children or partners who may act as primary stressors or perpetrators. Alternatively, the lower prevalence detected among the oldest age brackets may partially reflect a survival bias or a significantly reduced capacity to disclose abuse due to advanced physical frailty and cognitive vulnerabilities (Oyovwi & Oyovwi, 2024).

Depressive symptoms emerged as a robust independent correlate of EA, with symptomatic older adults exhibiting a 71% increase in the likelihood of reporting abuse. Although the cross-sectional design of the SABE Study precludes definitive causal inferences, prior longitudinal data strongly support a bidirectional relationship: depressive states may both increase interpersonal vulnerability—rendering an individual less capable of defusing conflict or managing caregiver stress—and act as a severe psychological consequence of ongoing chronic mistreatment (Yao, 2025). This powerful association reinforces the absolute necessity of integrating comprehensive mental health screenings into routine elder care programs, positionally leveraging psychosocial evaluations to detect early warning signs of domestic vulnerability (Dahal et al., 2021; Muhammad et al., 2021).

A particularly notable and nuanced finding of this study relates to the independent association between an Evangelical religious affiliation and higher odds of EA. Older participants identifying as Evangelicals were nearly twice as likely to report abuse compared to Catholics. Crucially, this relationship must not be interpreted as a simplistic causal link (Lechuga & Jones, 2024), but rather as a manifestation of a complex interplay of socioeconomic, structural, and socio-spatial dynamics. In Brazil, and particularly within the São Paulo macrometropolis, religious affiliation is deeply intertwined with territorial segmentation. Evangelical communities have experienced exponential demographic growth within urban peripheries and informal settlements. These regions are historically characterized by heightened social vulnerability, overcrowding, structural violence, and restricted access to state protective services—factors that collectively exacerbate domestic tension and interpersonal conflicts within the household (Py & Gracino Júnior, 2025; Araújo et al., 2021).

The importance of conceptualizing religious affiliation as a proxy for complex sociodemographic and territorial realities is further supported by the distinct prevalence rates observed across Protestant subgroups in the bivariate analysis. The variations between historical Protestants and Evangelical groups likely reflect deep internal heterogeneity. Historical Protestant denominations typically comprise older, socioeconomically established congregations occupying centralized urban strata, contrasted against the more recently established, peripherally located Pentecostal and Neo-Pentecostal movements.

Furthermore, sociocultural norms regarding religious authority, family preservation, and coping mechanisms exert a dual, paradoxical effect in these environments. On one hand, the dense social fabric and hyper-local presence of Evangelical churches may provide a highly supportive environment that empowers members to voice their grievances, perceiving the local church or pastoral circle as a secure, sanctified space for personal disclosure (Turhan, 2022). On the other hand, rigid or dogmatic interpretations of patriarchal authority and domestic endurance can act to justify controlling behaviors, minimize transgressions, or actively discourage external legal disclosure to preserve the sanctity of the family unit, establishing a structural "sacred silence" (Coelho-Júnior, 2022). This cultural complexity deeply shapes how domestic friction is conceptualized within the household—whether normalized as a form of pedagogical or corrective discipline against an individual deemed to have disrupted domestic order, or framed as an institutional cross to bear (Endler, 2025).

Interestingly, the absence of independent associations with individual-level variables such as education, income, and absolute living arrangements contrasts with parts of the international literature. This may reflect the homogenizing pressure of dense urban living conditions in South American metropolises. Large urban centers like São Paulo can blur individual economic differentials by exposing community-dwelling older adults to shared environmental stressors, high crime rates, and collective infrastructure deficits, irrespective of marginal variations in personal income (Drommi et al., 2025).

### Implications for Policy and Practice

These findings carry immediate practical implications for public health and social protection frameworks. Mandating the integration of standardized EA screening alongside depressive symptom monitoring within primary care and community-based health strategies (such as the Brazilian Family Health Strategy) could fundamentally optimize early detection rates. Recognizing the multifactorial etiology of EA, secondary interventions must simultaneously target individual mental health support, family conflict mediation, and culturally tailored community outreach (Storey, 2020). Specifically, training community health agents to recognize subtle psychosocial markers, combined with establishing formal channels of communication with faith-based organizations, can cultivate vital protective networks. Leveraging the significant community trust and moral authority possessed by local religious leaders can establish efficient secondary referral systems and educational platforms to actively dismantle domestic violence in aging populations (Brijoux et al., 2021).

### Limitations

The strengths of this study include its representative population-based design, the implementation of a culturally validated screening instrument, and the rigorous statistical adjustment for a wide array of potential confounding variables. To our knowledge, this is the first epidemiological study to document an independent association between elder abuse and Evangelical affiliation in a representative Brazilian sample.

Nevertheless, several limitations must be acknowledged. First, the cross-sectional nature of the data restricts any capacity to establish temporal sequencing or causal pathways. Second, despite the use of a validated scale, the reliance on self-reported measures introduces a risk of underreporting driven by cognitive barriers, social stigma, or fear of domestic retaliation. Third, because the SABE Study targets community-dwelling individuals exclusively, these results cannot be generalized to institutionalized or long-term care populations.

Finally, although our multivariate models were thoroughly adjusted for individual-level socioeconomic indicators (income and education), religious affiliation in this setting may still capture unmeasured neighborhood-level variances, territorial exclusion, or local crime rates. Future research utilizing georeferenced longitudinal models is critically needed to fully disentangle the spatial intersections between specific urban territories and the protective or risk-enhancing mechanisms of religious communities.

## Conclusions

In conclusion, elder abuse represents a significant, yet largely veiled, public health crisis within the rapidly transitioning Brazilian demographic landscape. The independent associations identified with depressive symptoms and Evangelical religious affiliation, alongside the heightened vulnerability observed among the younger-old, emphasize that vulnerability in late life is inherently multidimensional. Future longitudinal studies are imperative to map precise causal pathways and to rigorously evaluate the efficacy of community-led, person-centered interventions that effectively bridge mental healthcare, family support systems, and religious community engagement.

## Appendix

### Geriatric Mistreatment Scale Items

Response options: [] Yes [] No [] Don't know.

1. In the last year, have any of your friends or family shouted at you for no reason? *(verbal** abuse)*
2. In the last year, have any of your friends or family called you a name that you don’t like? *(Verbal abuse)*
3. “In the last year, have any of your friends or family touched or spent your money without your permission?”
4. “In the last year, have any of your friends or family threatened you because you did not do what they wanted?”
5. “In the last year, have any of your friends or family hit or slapped you?”
6. “In the last year, have any of your friends or family shaken you?”
7. “In the last year, have any of your friends or family stolen your money or something else belonging to you?”.
